# Ethical coaching and athlete transitions – A Foucauldian perspective on high-performance sports

**DOI:** 10.3389/fspor.2025.1675173

**Published:** 2025-10-29

**Authors:** Göran Gerdin, Richard Pringle

**Affiliations:** ^1^ Linnaeus University, Växjö, Sweden; ^2^ Universitetet I Innlandet, Elverum, Norway; ^3^ Monash University, Melbourne, VIC, Australia

**Keywords:** ethical coaching, Foucauldian theory, high-performance sport, athlete transitions, disciplinary power

## Abstract

High-performance sport is often celebrated for cultivating discipline, resilience and excellence. Yet, the same structures that produce elite performance frequently rely on disciplinary practices that can compromise athlete autonomy, well-being and identity development. This article interrogates the ethical dimensions of coaching in high-performance sport through a Foucauldian lens, drawing on concepts such as disciplinary power, technologies of the self and the aesthetics of existence. Building on the work of Jim Denison and colleagues, as well as our own previous work, we examine how coaching practices shape athlete subjectivities both during and after elite sporting careers. The paper presents a framework that coheres a number of key concepts from existing coaching research and enriches them through a Foucauldian ethical perspective, offering a unified way of understanding the ethical dimensions of coaching. We argue that coaching must be reimagined as an ethical, relational and reflexive practice that goes beyond harm reduction to actively support athlete well-being and meaningful, sustainable transitions beyond sport. We explore how ethical self-creation can enable coaches to resist dominant norms and develop care-based coaching approaches that challenge the performance-at-all-costs ethos. We also consider how these insights align and contrast with existing youth sport philosophies and conclude by proposing a set of guiding principles for fostering ethical and sustainable coaching environments. In doing so, the paper offers a contribution to sport coaching research and practice by illuminating how coaches can engage in ethical self-work and systemic transformation, positioning athletes not only as performers but as whole persons capable of living meaningful lives in and beyond sport.

## Introduction

High-performance sports coaching operates within a complex landscape where the pursuit of excellence often comes at a significant personal and ethical cost to both athletes and coaches. Within this environment, disciplinary practices such as surveillance, control and normalization have become standardized, shaping not only athletic performance but also athlete subjectivity. These practices frequently prioritize winning and compliance over well-being and autonomy, raising critical questions about what it means to coach ethically in high-performance sport. As Jones et al. (1) have previously argued, despite mounting concerns about athlete welfare, the high-performance sport system remains dominated by coercive power relations that are often concealed behind discourses of professionalism and personal responsibility. These dynamics contribute to athletes being expected to internalize discipline, self-regulate emotions and behaviors and manage success and failure as private matters. Such conditions can lead to anxiety, disconnection and emotional exhaustion, particularly when athlete identities are closely tied to performance metrics [see also (2–5)]. Denison and Avner (6) similarly argue that prevailing coaching methods often rely on disciplinary techniques that render athletes compliant and manageable, but in doing so, they risk suppressing critical thought and ethical development. Drawing on Foucault, they suggest that coaches should not merely focus on controlling athlete behaviour but should instead foster environments that encourage ethical reflection, autonomy and the development of more expansive athletic subjectivities.

Previous research has further highlighted that the relentless pursuit of excellence in sport can carry substantial personal and ethical costs for both athletes and coaches. These costs are not only physical but psychological, emotional and relational. For athletes, highly controlling coaching environments have been associated with reduced autonomy, diminished motivation and increased risk of burnout and ill-being (7–9). For coaches, the constant pressure to produce results can compromise their capacity to care and reflect, often leading to moral distress, relational strain and a narrowing of pedagogical repertoires (10, 11). These findings indicate that the high-performance sport system is not neutral; it actively shapes the subjectivities of those within it, often privileging control and compliance at the expense of autonomy, care and ethical responsibility.

In this paper, we build upon our previous work and the Foucauldian scholarship of Jim Denison and colleagues to interrogate the ethical and power dimensions of coaching and athlete transitions. Drawing on Foucault's concepts of disciplinary power, technologies of the self and the aesthetics of existence (12–14), as well as narrative accounts and critical scholarship, we examine how dominant practices in high-performance sport shape athlete identities in ways that can hinder autonomy and make transitions beyond sport challenging. We also consider how moments of disruption and reflection can open space for alternative coaching practices that emphasise care, relationality and holistic development. Particular attention is given to how Foucauldian ethics can be brought into dialogue with established youth coaching philosophies to reimagine coaching as an educational and ethical practice. Rather than offering prescriptive models, we invite coaches and practitioners to critically reflect on their roles in shaping the ethical landscapes of high-performance sport.

In doing so, the paper also contributes conceptually by presenting a framework that brings together key concepts from existing coaching research and enriches them through a Foucauldian ethical perspective. This approach offers a unified way of understanding the ethical dimensions of coaching and positions Foucauldian ethics as a conceptual lens through which these ideas can be critically examined and extended.

## The ethical dilemmas of high-performance sports, athlete transitions and the problem of docility

The current high-performance sports culture frequently valorizes winning at all costs, often at the expense of athletes’ physical and psychological health (15–17). This culture is sustained by disciplinary practices that emphasize surveillance, control and compliance, practices that are normalized through coaching methods and institutional expectations (18). Athletes are trained to regulate themselves according to external standards of success, often internalizing notions of mental toughness and self-sacrifice as essential virtues. These environments do not merely produce athletic performance. They shape the athlete's subjectivity, often rendering them what Foucault (19) termed “docile bodies”; individuals conditioned to conform, submit and perform according to externally imposed norms. Despite these risks and pressures, many athletes remain deeply invested in high-performance sport. As recently argued by Jones et al. (20), athletes are not simply passive recipients of discipline. They also derive purpose, belonging and identity from the structures of elite sport. The same systems that contribute to injury, anxiety and disordered relationships to the body can also produce moments of joy, pride and connection (20). This paradox speaks to the complexity of ethical life in sport. The very environments that constrain athletes can also be deeply meaningful, which makes the work of ethical critique both more difficult and more necessary.

Coaches play a pivotal role in either perpetuating or challenging this culture. Previous work [e.g., (18)] reveals how the disciplinary techniques employed by coaches work to produce docile athletes, aligning with Denison and Mills (16), who describe the normalization of compliance and the control over athletes’ bodies and behaviours within disciplinary frameworks. Athlete identities and well-being are thus subordinated to the imperatives of performance and competition, where conformity, sacrifice and control are rewarded. These practices have been shown to contribute to a range of issues: burnout, chronic injuries, disordered eating and mental health struggles including anxiety, depression and a loss of self-worth (15). As Denison et al. (17) have observed, the disciplinary legacy of coaching often normalizes extreme control over athletes’ bodies and minds. This aligns with Barker-Ruchti et al.'s (21) critique of high-performance environments that silence and disempower athletes, reinforcing cultures where obedience and dependency are framed as essential for success and, at its worse, producing a context that allows for the abuse of athletes (22). Under such conditions, autonomy and critical reflection are actively suppressed and athletes learn to regulate themselves in ways that maintain existing power structures.

The transition out of elite sport is widely recognised as one of the most challenging phases in an athlete's life. This period often lays bare the long-term effects of disciplinary power and its impact on identity, autonomy and well-being. Former athletes frequently struggle to reimagine themselves outside the structured and regulated environments of high-performance sport, where their lives were governed by intensive training routines, external validation and constant surveillance (1, 20). As Denison et al. (17) argue, the creation of such docile athletes may facilitate short-term compliance and competitive success, but it undermines athletes’ capacity for critical agency and self-directed decision-making over the long term. Athletes who have been trained to equate obedience with success may find themselves unable to navigate life after sport, especially when faced with the ambiguity, freedom and unstructured nature of everyday contexts. As Coakley (15) and Stambulova et al. (23) note, transitions from elite sport are rarely smooth, and the risk of psychological distress, disorientation and even existential crisis is heightened when athletes’ identities have become narrowly defined by performance outcomes.

Recent work by Jones et al. (20) builds on Foucault's (24) concept of “confessional techniques” to show how former athletes internalise disciplinary norms well into retirement. Their study of retired British footballers illustrates how athletes maintain a confessional mode of self-relation, one characterised by continuous self-monitoring, self-blame and self-correction. Athletes spoke of never “being good enough” (p. 12) or being turned “into a tough- and thick-skinned human” (p. 13), even years after leaving professional sport. This ongoing self-surveillance was tied to a deeper, learned docility, where the norms of high performance continued to shape their everyday lives. In many cases, these individuals praised the discipline and resilience developed through sport, crediting it for their subsequent success in employment or education. Yet what appears as adaptability may also signal a troubling readiness to comply, to please and to avoid critical interrogation of norms. In this way, disciplinary power extends beyond the sport setting, shaping athletes into ideal neoliberal subjects: self-regulating, efficient, productive and emotionally contained.

This paradox presents a significant ethical dilemma for coaching. Should high-performance sport be praised for producing “life skills” or critiqued for cultivating compliance and suppressing autonomy? While some former athletes may thrive in post-sport contexts, others suffer from anxiety, loss of identity or an inability to define themselves outside of sport (1, 23). These difficulties are often attributed to inadequate career planning or poor exit support, but such explanations risk overlooking the deeper disciplinary structures that shaped the athlete's identity in the first place.

Building on these insights, we argue that coaches have a transformative role to play in preparing athletes not only for competition but also for life beyond sport. We recognise, however, that this might be difficult to achieve in practice. Many coaches do not see themselves as life coaches or as agents of social transformation, nor are they always equipped or supported to take on such roles. Ethical coaching must nonetheless move beyond short-term performance enhancement or even harm reduction. It should foster long-term autonomy, resilience and critical self-awareness. This requires a conscious departure from conventional disciplinary techniques and an investment in athlete-centred, ethically reflective coaching. Drawing on Foucault's ethics of self-creation (12–14), we propose that coaches integrate opportunities for athletes to reflect critically on their experiences, beliefs and values during their careers, not only at the point of retirement. This reflective practice can challenge the confessional mindset and help athletes develop alternative modes of subjectivity not solely tied to performance metrics or disciplinary logics. As Denison et al. (17) suggest, this work must be relational and situated, taking into account the power dynamics between coaches and athletes and recognising the responsibility that coaches carry in shaping ethical and sustainable sporting environments.

Ethical coaching for transitions does not imply that all athletes must leave sport critically awakened or politically radicalised. Rather, it calls for environments where alternative identities and futures are made imaginable. This includes transforming the coach-athlete relationship from one defined by regulation and compliance to one grounded in care, reflection and the shared task of creating meaningful lives both within and beyond sport. Such a shift in orientation demands more than individual goodwill. It requires coaches to critically reimagine their practices through an ethical lens, one that foregrounds care, reflexivity and transformation (17). In the following section, we explore how Foucauldian ethics, particularly the concept of the aesthetics of existence, offers a theoretical and practical pathway for cultivating coaching practices that challenge disciplinary norms and support athletes’ holistic development in ways that extend beyond performance.

## Foucauldian ethics in coaching as a tool for ethical coaching

Foucault's ethics of self-creation offers a powerful conceptual framework for critically reimagining coaching practices in high-performance sport (12, 13). Central to this ethics is the notion of the “aesthetics of existence,” a call for individuals to craft their lives as works of art through reflective engagement, ethical self-formation and transformation (12, 14). This perspective prioritizes critical awareness over compliance and positions ethical subjectivity as an ongoing practice of questioning and becoming. In the realm of coaching, this implies an active interrogation of normalized practices and the development of alternatives that center athlete well-being and holistic growth.

Foucauldian ethics does not prescribe a universal code of conduct but instead calls for an ethics grounded in situated self-reflection. For coaches, this means engaging in practices that challenge the taken-for-granted disciplinary logics of high-performance sport. Rather than producing athletes who are merely compliant and efficient, coaches informed by Foucauldian thinking aim to foster spaces where autonomy, creativity and ethical decision-making are possible. Denison and Avner (6) argue that coaches must recognize their complicity in sustaining power relations and begin to resist coaching strategies that reproduce docile athletes. This requires moving beyond instrumental notions of success to consider how coaching practices shape subjectivities and life trajectories.

An illustrative example of this is provided in Gerdin et al. (18), where Gerdin reflects on his own trajectory as an elite junior tennis coach. Faced with the suicide of a young athlete, he was forced to confront the limitations and ethical consequences of his former coaching methods, which had emphasized discipline, control and performance above all else. This moment of crisis catalyzed a profound re-evaluation of what it meant to be a coach. Drawing on Foucault's concept of self-work, Gerdin described a shift from authoritarian, performance-driven coaching to an approach that centered enjoyment, personal growth and athlete resilience. As he explains, “I could not help but question everything that I had done as coach and even my involvement in sport and professional coaching” [(18), p. 33].

Such a transformation is emblematic of Foucauldian ethics in practice. It involves not only a change in behavior but a fundamental reorientation of how power is exercised and how relationships are structured. Through the practice of “parrhesia” (“truth-telling”) coaches can begin to speak and act against dominant discourses that prioritize winning over well-being. Gerdin's narrative also illustrates how ethical coaching is not a destination but a process. The work of becoming an ethical coach requires continuous self-examination, discomfort and openness to transformation. As Denison and Mills (16) contend, ethical coaching cannot be reduced to a checklist of actions but must emerge through an ongoing engagement with the question of how to live and act otherwise.

In this light, Foucauldian coaching is inherently relational. It recognizes that athletes are not passive recipients of knowledge or training but active participants in shaping their own subjectivities. Coaching thus becomes a collaborative practice where both coach and athlete engage in processes of ethical self-formation. This calls for the cultivation of dialogical relationships, where listening, empathy and mutual respect replace coercion and control. Foucault's ethics also invites coaches to consider how their own histories and desires shape their practices. As Gerdin et al. (18) acknowledge, the transition to ethical coaching is not easy. It often stems from an incident or via a cumulative epiphany that spurs critical reflection and entails confronting one's complicity, re-evaluating personal investments in success and grappling with institutional pressures. Yet it is precisely through this confrontation that ethical possibilities emerge. By viewing coaching as an aesthetic and ethical practice, grounded in care and critique, coaches can create spaces that support the holistic development of athletes both within and beyond sport.

Narrative inquiry provides a powerful methodological and pedagogical framework for examining, understanding and transforming coaching practices. Rooted in the interpretive and critical traditions, narrative inquiry enables coaches to explore the complexity of their practice through stories of lived experience, thereby offering insights into the ethical, emotional and relational dimensions of sport. As Gerdin (25, 26) and others have demonstrated, narratives of self-reflection allow coaches to critically engage with their actions and the broader cultural logics that shape those actions. By foregrounding lived experience, narrative inquiry facilitates a deeper examination of how coaching identities are formed, how ethical dilemmas are encountered and negotiated, and how change might emerge through critical self-work. Narratives serve as more than just accounts of past events. They are performative and constitutive, shaping the ways in which coaches make sense of themselves and their ethical responsibilities. As Coakley (15) has argued, sport is often embedded with cultural assumptions that valorize competition, toughness and success, frequently at the expense of well-being and critical thinking. Narrative inquiry makes visible these cultural assumptions by inviting coaches to reflect on the tensions they experience between institutional expectations and personal values. In doing so, narrative inquiry not only produces knowledge about coaching but becomes a vehicle for ethical transformation.

For instance, Gerdin's autobiographical narratives of coaching elite junior tennis players illustrates how moments of personal crisis can prompt a re-evaluation of coaching philosophies and practices (18). His reflections on the emotional and ethical toll of authoritarian, performance-driven methods led to a shift toward a coaching approach grounded in care, joy and holistic development. Such narratives illuminate the tensions inherent in being both a critic and a participant within high-performance sport. They reveal the difficulty of resisting normalized practices tied to pleasure, power and prestige, even when those practices conflict with one's ethical commitments. Gerdin's account highlights how narrative inquiry can be used not only to document these tensions but to actively work through them. As Barker-Ruchti et al. (21) notes, stories of ethical struggle and transformation challenge the idea that coaching is value-neutral and instead position it as a site of ongoing negotiation over what constitutes good practice. Importantly, narrative inquiry can foster collective reflection. By sharing stories of ethical dilemmas, moments of uncertainty and changes in practice, coaches can open up spaces of dialogue that disrupt the isolation often experienced in high-performance environments. This collective dimension of narrative inquiry can support the development of communities of practice that are committed to more just and caring approaches to coaching. In this way, narrative inquiry does not offer a formula or checklist for ethical coaching but cultivates the disposition and skills necessary for critical reflection, relational awareness and ethical responsiveness.

Gerdin (26) further elaborates on this in his autoethnographic account of navigating the “liminal spaces” of high-performance coaching, where he documents the ambiguities and contradictions of trying to be both a critical pedagogue and an embedded practitioner. As he reflects, “this aesthetics of existence … is not an easy or linear process. It is a process filled with conflicts and tensions where sort of a recurrent elite athlete identity is still drawing me back to my former elite athlete self” (p. 29). These insights underscore the affective weight and institutional complexity involved in ethical coaching. His use of narrative opens up possibilities for coaches to see themselves not just as executors of a system but as subjects capable of agency and ethical transformation.

Narrative inquiry, then, is not just a method of research but a mode of ethical engagement. It aligns closely with Foucauldian notions of ethical self-creation, where the subject is not passively shaped by external norms but actively involved in the formation of their own moral and relational orientations. As such, narrative inquiry can offer both a critique of dominant coaching paradigms and a pathway toward reimagining coaching as an ethical, reflexive and relational practice. In doing so, it contributes to a broader vision of sport where the well-being, autonomy and holistic development of athletes are not secondary to performance but integral to what it means to coach well.

In the following section, we explore how these ideas align and contrast with youth coaching philosophies and what a Foucauldian-informed approach might offer in comparison.

## Foucauldian ethics in coaching: A comparison with philosophies of coaching youth

In reflecting on a Foucauldian-inspired approach to coaching, we recognised some apparent similarities to existing youth coaching philosophies [e.g., (27)]. Given these similarities, we examine the following question: what can we learn about ethical coaching by comparing a Foucauldian perspective with youth-focused coaching approaches?

Scholarly interest in the ethics of coaching children developed in the 1980s in response to the rapid increase in organised sport for children and growing concern for their wellbeing (28). One key philosophy emerging from this discourse is a player-centred approach often summarised as “*Children first: Winning second*”. This philosophy was initially promoted by Rainer Martens (29), founder of Human Kinetics and the American Coaching Effectiveness Program, in opposition to elite sport paradigms that emphasised winning above all else. Martens warned that an excessive focus on winning could have adverse impact on young people and detract from what was best for their development and enjoyment. He argued that adults carry an ethical responsibility, grounded in a form of virtue ethics, to care for children in ways that support their holistic growth physically, affectively, socially and cognitively, while promoting life skills such as teamwork, resilience, cooperation and communication.

Martens’ approach shares common ground with our Foucauldian inspired approach above and with Tinning's (30) argument that coaching is fundamentally an educational endeavour imbued with ethical responsibility. Martens’ underpinning youth-philosophy does not dismiss the importance of winning. He acknowledged that, in some instances, focusing on winning could be beneficial for individual children. However, his broader philosophical approach encourages coaches to critically examine how the pursuit of winning can either enhance or undermine young people's wellbeing and development. In this light, a youth coaching philosophy promotes reflective practice, urging coaches to consider the consequences of their methods and actions. Relatedly, we argue that critical reflection is not only essential for coaches but is also a key life skill that young athletes should learn. Coaches should support young people, accordingly, in reflecting on their sporting experiences and help them develop the complex skill of critical reflexivity.

Tinning (30) noted that critical reflections can address technical, practical or even emancipatory aspects of sport participation. He suggested that if a coach aims to foster emancipatory outcomes, they should adopt coaching styles aligned with critical pedagogical approaches, akin to those used in educational contexts. At the same time, Tinning recognised that adopting such an approach is not a requirement in sport and depends on each coach's values and aims. Nevertheless, we argue that young athletes should be encouraged not only to reflect on their performance, training, or decision-making within sport but also on their broader experiences, both positive and negative. This form of reflection can support greater self-awareness about the costs and benefits of sporting involvement. For some, this may possibly lead to the decision to withdraw from sport; for others, it may reinforce their commitment. Importantly, the process of critical reflection is not about achieving a specific outcome (31). Rather, it is valuable in and of itself, particularly when it fosters athletes’ capacity for critical reflexivity and self-knowledge.

Foucauldian coaching scholars have expressed similarly concerns about the costs associated with the pursuit of winning, particularly in relation to the disciplinary technologies employed by coaches that can negatively impact athletes’ long-term wellbeing. Markula and Pringle (32), for example, stated that “Within the sociological study of sport and exercise, a number of scholars … illustrate how sport and exercise programmes discipline and normalise participants to render their conforming but biomechanically or physiologically efficient bodies ‘docile’” (p. 45). Yet this does not mean that a Foucauldian approach regards disciplinary techniques as inherently good or bad.

In contrast, Foucault recognised that disciplinary powers can produce both positive or negative outcomes. In a rare moment of self-reflection, Foucault (33) recognised that, as a hard-working scholar he too had been subject to the disciplinary technologies embedded in academia. He stated that he had “worked like a dog” [(33), p. 131] throughout his academic life not because he was “interested in the academic status” (p. 131) that he could gain but as a technique for self-transformation. Foucault explained that he had a personal investment in each of the social problems that he explored and that his “hard work” induced critical self-reflection that allowed for possibilities or self-change: “For me, intellectual work is related to what you could call “aestheticism,” meaning transforming yourself ….This transformation of one's self by one's own knowledge is, I think, something rather close to the aesthetic experience” [(33), p. 131]. In this light Foucault viewed the disciplinary practices that produced him as a hard-working scholar in a positive manner, as he was not naively controlled and managed by those disciplinary powers but he had critically reflected on what they were producing and how, most importantly, they allowed for transformational self-changes. Foucault (34) referred to this ability to actively shape one's subjectivity *within* the workings of power, as a “practice of freedom” and he considered this practice as an ethical task: “Freedom is the ontological condition of ethics. But ethics is the considered form that freedom takes when it is informed by reflection” (p. 284).

Foucault's aim in examining the workings of disciplinary technologies, relatedly, was to not to say that they are inherently problematic but to make people *aware* of how these forms of power can operate insidiously and, at times, with negative consequences. Hence, his aim was to promote critical awareness of how these forms of power operate. In a similar manner, Markula and Pringle (32) stated that their aim in examining sport as a disciplinary institution was also to raise awareness of these forms of power embedded within sport and the associated ethical responsibilities of working within sport and exercise realms:

We can understand how the sport and fitness disciplines are an integral part of the workings of disciplinary power in contemporary societies. Yet, more importantly, it can be recognised that ‘we’ are all active participants in numerous relationships of power with respect to sport and fitness practices, and that the combined total of these power relations produces the overall shape of sport and fitness practices. A responsibility stems from this recognition, a responsibility that invites us to negotiate our various power relations with a sense of ethics and a desire to minimise harmful modes of domination. (p. 45)

In contrast to the youth sport philosophy of “Children first, winning second”, a Foucauldian-influenced coaching philosophy might be framed as “Critical awareness of power relations and ethics first, winning second”. Both traditions share a concern with athlete wellbeing and reject the notion that performance outcomes alone define coaching success. Both also affirm that disciplinary or training practices are not inherently harmful but must be examined critically and used with ethical intent. Drawing on these insights, we suggest that coaches working in elite sport might adopt the guiding youth coach philosophy of “Athletes first: winning second”. This approach foregrounds an ethics of care, supports athlete autonomy and promotes critically reflexive practice attuned to each athlete's unique needs, experiences and long-term development.

The topic of athlete autonomy has been recognised by various researchers as an important and interconnected aspect of effective coaching [e.g., see (8, 9)]. Autonomy is identified by Deci and Ryan (35, 36) as one of the three basic psychological needs for athletes, along with competence and relatedness. They argued that when a coaching style increases an athlete's sense of autonomy, this enhances their motivation, resilience and commitment.

The importance of “care” within coaching relationships has also been examined by several researchers [e.g., (10, 11, 37)]. Cronin (11) advocated that the athlete-coach relationship should be conceived as a caring relationship in which coaches support athletes’ sporting needs. He also recognised that “extant studies have often used coaches’ reports to define the needs of athletes and authoritatively describe what actions are caring” (p. 2). The voices of athletes, with respect to their needs, have accordingly been marginalised within the research literature. Cronin, in turn, argued for recognising that coaches and athletes exist within a relationship and that “caring”, as such, should be considered as “relational” (p. 2). Moreover, within this relationship, both coach and athlete should have the freedom and ability to express their needs.

Cronin's (11) work on the relational aspects of care in coaching has considerable overlap with Foucault's work on relationships of power. Foucault (19) considered power as a relationship between “free” people, who have the ability to express ideas and, if needed, the ability to resist. He did not view relationships of power, including those within coaching, as inherently positive or negative. However, he was concerned with forms of domination that “stem from specific relationships of power and act to limit the field of possible actions for some individuals or groups of people” [(32), p. 38]. In this respect, Foucault (38) was interested in individuals developing their own techniques of management, ethics and practices of self to “allow the games of power to be played with a minimum of domination” (p. 18). In the following sections, we discuss how coaches can develop their own techniques of management and ethical practices of self.

## Toward ethical and sustainable coaching practices

To advance ethical and sustainable coaching practices in high-performance sport, we propose a multi-faceted approach grounded in critical reflection, ethical self-work and systemic transformation. This approach is informed by Foucault's ethics of self-creation (12–14, 33, 34), Denison and Mills’ (16) critique of disciplinary coaching practices and narrative research that foregrounds the relational and emotional dimensions of ethical coaching (18, 21, 26). Together, these perspectives point to the growing importance of coaching that supports athlete development and well-being beyond short-term performance metrics. In the following we offer potential strategies for coaching ethically.

### Problematizing norms

Ethical coaching begins with the capacity and willingness to critically examine the dominant assumptions and values underpinning high-performance sport. Coaches should reflect on beliefs such as the centrality of winning, the normalization of suffering, and the idea that discipline and sacrifice are always virtuous. These are not neutral assumptions, but products of historically embedded power relations that determine what is seen as legitimate or valuable. Foucault (33) reminds us that disciplinary techniques have no inherent moral value; their effects and consequences must be interrogated. His notion of a “critical ontology of ourselves” [(39), p. 50] encourages continuous questioning of how our identities and practices are shaped by power, and how we might act differently. Coaches should therefore reflect not just on what they do, but why they do it and to what end. This involves examining how norms like mental toughness and competitive intensity shape training environments and potentially cause harm alongside growth. Asking difficult questions such as “Whose interests do these methods serve?” or “What kinds of people are we producing?” opens space for alternative, more ethical coaching possibilities. Problematizing norms is not about abandoning ambition but reimagining coaching as a reflective and socially responsible practice that considers both individual flourishing and collective values.

### Fostering athlete autonomy

A central aspect of ethical coaching is supporting athlete autonomy not just by offering choice within narrow limits but by creating environments where athletes can reflect, exercise agency and shape their experiences. Autonomy is not a fixed trait but emerges relationally through trust, dialogue and meaningful engagement. Foucault's (12, 14) ethics of self-creation helps us understand this process as one of ethical formation, where individuals critically engage with their values, desires and actions. Coaches play a vital role in enabling this by stepping away from authoritarian models and instead inviting athlete input, experimentation and questioning. Small practices, such as co-designing training sessions or discussing the purpose behind drills, affirm the athlete as a thinking and feeling subject. As Gerdin (26) argues, treating athletes as active participants in their own subject formation, rather than as passive recipients of instruction, fosters reflective engagement and supports development that extends beyond sport. Encouraging autonomy also requires acknowledging the diverse values and goals athletes bring to sport and adapting practices accordingly. This is not about dismissing structure or discipline, but about applying them to support holistic growth. Ethical coaching, as Denison and Mills (16) note, involves nurturing individuals capable of self-understanding and critical thinking. Autonomy-focused coaching challenges control-based systems, asking coaches to listen more deeply and recognise athletes as persons undergoing complex processes of becoming.

### Promoting holistic development

Ethical coaching prioritizes the holistic development of athletes as whole persons—not merely performers. This includes addressing their emotional, social, cognitive and ethical needs alongside physical training. Coaching should support athletes in ways that help them flourish during and after their sporting careers. Foucault (12, 14) framed ethics as a practice of freedom, involving care for the self and critical engagement with the world. Coaching, therefore, becomes more than instruction; it is a space for mutual growth. Gerdin et al. (18) and Gerdin (26) show how a shift from control-focused coaching to relational care can support this broader development. Success must be redefined beyond medals or rankings to include traits like empathy, resilience and ethical reasoning. Martens (28) and Tinning (30) emphasize that coaching is an educational practice and should cultivate wide-ranging human capacities. Coaches must consider how their practices affect athletes’ sense of self and relationships with others. Barker-Ruchti et al. (21) highlights how team dynamics can either support or harm well-being. Athletes are more than their roles in sport; they are people with complex lives, and coaching should support their ability to navigate transitions and challenges. Promoting holistic development is not an extra but a foundational commitment, ensuring that sport contributes positively to life beyond performance.

### Engaging with ethical self-work and narratives

Ethical coaching is grounded in continuous self-work. Engaging with personal and shared narratives helps coaches reflect on the ethical dimensions of their practices and how power operates in their environments. Rather than abstract principles, narratives can provide context-rich insights into the moral challenges coaches face. Gerdin et al. (18) shows how moments of personal crisis (like an athlete's suicide) prompt critical reevaluation of coaching practices. These stories encourage coaches to examine complicity in systems that may cause harm and to consider alternative, more caring ways of working. Denison et al. (17) argue that such reflection is key to ethical practice. Narratives not only document experiences but act as “parrhesia” (“truth-telling”) that can challenge harmful norms. When shared in communities of practice, these narratives invite dialogue and disrupt the isolation of ethical reflection. Barker-Ruchti et al. (21) highlights the emotional and relational stakes of coaching, reminding us that ethical coaching is inseparable from how we relate to others. Stories of care, failure and change are not ends in themselves but serve as catalysts for action. Engaging with narratives cultivates attentiveness, humility and critical reflexivity, all vital for ethical coaching. Coaches must remain open to examining how they are shaped by sport's cultures and how they might work to reshape them in turn.

### Advocating for systemic reform

Ethical coaching cannot rest on individual goodwill alone; it requires systemic reform. Coaches operate in structures that often reward performance above well-being and discourage care-based practices. These environments must be transformed to make ethical coaching viable. Denison et al. (17) and Gerdin et al. (18) highlight how institutional pressures can force coaches to prioritise winning even when they wish to act differently. Changing this requires revisiting how success is defined, how policies are written, and how coach education is structured. Supporting athlete-centred models, creating post-career pathways and valuing holistic development are essential steps. Systemic reform should include mentoring and reflective spaces for coaches, as well as institutional incentives that align with ethical principles. Drawing on Foucault (12, 33), we must also recognise that power is embedded in daily practices. Change happens not just through policies, but through shifts in culture and relationships. Everyone in sport, coaches, athletes, administrators and researchers, has a role in shaping norms. Reform, then, is both structural and cultural. It means creating conditions where care, reflection and autonomy are part of the everyday. Ethical coaching should not be an exception but the standard, supported by systems that affirm human dignity and development over mere performance.

Taken together, these five dimensions - problematizing norms, fostering athlete autonomy, promoting holistic development, engaging with narratives and advocating for systemic reform—constitute an interconnected framework for ethical and sustainable coaching. They reflect a shift from viewing coaching as a purely technical endeavour focused on outcomes to understanding it as an ethical and relational practice that shapes subjectivities, identities and futures. Grounded in Foucauldian notions of ethical self-creation and care for the self and others, this framework encourages coaches to move beyond compliance with inherited norms and toward the cultivation of reflective, responsive and just practices. Importantly, these changes cannot rest on individual will alone. They require collective action and institutional commitment to reimagining what coaching can be, so that both athletes and coaches may flourish not only in sport but also in life beyond it.

## Conclusion

High-performance sports coaching is increasingly subject to ethical scrutiny. While traditional disciplinary practices have contributed to elite performance, they have also been shown to produce harmful consequences for athletes’ well-being, autonomy and long-term development (1, 16). Drawing on Foucauldian ethics, narrative inquiry and critical sport scholarship, we have proposed a framework for reimagining coaching as an ethical and relational practice. This involves shifting attention from short-term performance gains toward the cultivation of critically reflective, resilient, and self-aware individuals (17).

Rather than suggesting that high-performance sport is in crisis or must abandon its traditions entirely, we argue that there is value in examining how dominant practices shape athletes’ lives both within and beyond sport. As Markula and Pringle (32) remind us, disciplinary power is not inherently negative, but it carries ethical implications that must be considered. Coaches are therefore uniquely positioned to reflect on and potentially transform the environments they help to create. Through ethical self-work and sustained critical reflection, coaching can become a practice that not only supports sporting excellence but also prepares athletes for meaningful lives after competition (1, 6).

We recognise that despite the good intentions of coach researchers to promote athlete wellbeing or change pedagogical approaches, their actual influence remains limited (40). Cushion, Armour and Jones (41) highlight that the primary source of knowledge for coaches, stem from experience (i.e., how they were coached) and the observation of other coaches. We are accordingly aware that our aim within this paper to encourage coaches to place ‘athletes first and winning second’ will likely run counter to what many coaches value in the context of elite sport and that our practical impact may be limited. Therefore, the gap between coaching research and practice remains an issue worthy of further exploration (40). Yet we also remain steadfast in our belief that researchers should continue to advocate for certain ethical or pedagogical approaches within coaching. It is through the process of challenging taken-for-granted assumptions and encouraging critical reflection that the field of coach education will continue to develop. More broadly, the paper has sought to bring existing coaching research into dialogue with a Foucauldian ethical perspective, offering a way to unify and enrich established concepts through a critical lens.

Moreover, calls for inclusion, equity and social responsibility in sport do not imply that existing models have failed altogether. Rather, they reflect broader societal conversations about the values that should underpin institutions with significant cultural influence. Sport, as a powerful social institution, has the capacity to both reflect and shape social norms. Engaging more consciously with values such as care, autonomy and justice enables coaches to contribute to sporting environments that are not only competitive but also sustainable and life-affirming (17). By linking everyday coaching practices with broader ethical questions, this paper has argued for a coaching philosophy grounded in care, critical awareness and the shared task of human development. Coaches who embrace this approach may not only support athletes in their pursuit of excellence, but also in becoming reflective, autonomous and ethically engaged individuals, capable of thriving both within and beyond the world of sport.
